# Validation and Extension of the School Mental Health Self-Efficacy Teacher Survey

**DOI:** 10.1007/s12310-025-09807-5

**Published:** 2025-09-16

**Authors:** Simon Daniel, Heather L. McDaniel, Michael D. Lyons

**Affiliations:** 1 School of Education and Human Development, University of Virginia, Charlottesville, VA 22904, USA; 2 McCausland College of Arts and Sciences, University of South Carolina, Columbia, SC, USA

**Keywords:** School mental health, Self-efficacy, Validity, Implementation

## Abstract

As researchers identify methods for addressing schools’ capacity for implementing school mental health (SMH) services, there arises a need for psychometrically sound assessments of teacher self-efficacy for implementing SMH services. The current study provides validity evidence regarding the internal structure of the *School Mental Health Self-Efficacy Teacher Survey* (SMH-SETS) in the context of middle schools preparing to implement the *Early Identification System* (EIS), a universal mental and behavioral health screening checklist. We also explored convergent associations between the SMH-SETS and three EIS intervention reaction measures (i.e., perceptions of acceptability, appropriateness, and feasibility). Middle school teachers (*N* = 411) from southern and midwestern US districts participated in the study. Theoretically driven unidimensional, two-factor, and five-factor structures of the SMH-SETS were assessed. A five-factor structure was determined to yield the best fit to the data and factor scores were extracted for subsequent analyses. A “Support” factor relating to school-family collaboration and mental health promotion was significantly and positively associated with participants’ feelings that the EIS was acceptable (β = .46), appropriate (β = .39), and feasible (β = .42). A “Crisis” factor relating to mental health crisis response was significantly and negatively associated with participants’ acceptability (β = −.32) of the EIS. The multifactor structure supports a more nuanced view of SMH self-efficacy and may allow for greater ability to measure specific facets of SMH self-efficacy. Furthermore, the relationship between certain factors and intervention reaction measures may be of interest as researchers develop and assess practices to support SMH implementation.

## Introduction

The mental health needs of youth around the world drastically increased during the COVID-19 pandemic (Racine et al., 2021) and, in the United States, rates of youth mental health problems remain high (CDC, 2024). Although schools are often identified as having an important role in the mental health of youth (CDC, 2024), not all teachers are equipped to effectively address the mental health needs of their students (Reinke et al., 2011). Roughly half of public schools report that they cannot adequately provide mental health services to their students (National Center for Education Statistics [NCES], 2022). Limited capacity of schools paired with high youth mental health needs have prompted school leaders and researchers to consider novel ways to support effective implementation of school mental health (SMH) services. One way to bolster schools’ capacity to address youth mental health concerns is through the professional development of teachers. Professional development exercises (e.g., training, coaching, consultation, etc.) often target teachers’ knowledge, skills, and confidence to respond to youth mental health concerns. Self-efficacy is a construct that involves an individual’s self-assessed confidence to complete a task, and as a result, is a proximal outcome targeted in studies of SMH professional development. Thus, validated instruments measuring self-efficacy to implement SMH services are necessary for researchers, policymakers, and practitioners to understand the effects professional development has on teacher outcomes. This study addresses gaps in measuring teacher SMH self-efficacy by providing additional validity evidence for an existing measure of teacher self-efficacy to implement SMH services, the *School Mental Health Self-Efficacy Teacher Survey* (SMH-SETS; Brann et al., 2021), in a sample of middle school teachers. Specifically, we aim to provide additional evidence of the internal structure of the SMH-SET through confirmatory factor analysis and evidence of convergent validity through correlations of the SMH-SET scores with other related constructs. In doing so, the results may inform how future studies conceptualize and measure teacher SMH self-efficacy.

### Implementation of School Mental Health

Implementation frameworks, such as the *Consolidated Framework for Implementation Research* (CFIR; Damschroder et al., 2022), provide a roadmap for investigating implementation challenges and understanding characteristics that are associated with implementation success. The CFIR model has been used to determine environmental factors that affect SMH implementation, and the characteristics of school staff is one of the focal factors in the CFIR model. For example, a recent scoping review focused on teacher-led SMH efforts found that teachers’ beliefs, attitudes, self-efficacy, and training in implementing SMH programs were key for successfully implementing SMH services. The same review found that increasing teacher-level competencies through training and professional development served as effective facilitators of SMH implementation (Roshan et al., 2024).

The primary method of influencing the individual-level of SMH implementation is through professional development and training (Owens et al., 2014). Professional development serves the purpose of equipping implementers with the knowledge and competencies to deliver an intervention (Owens et al., 2014) and can bolster certain psychological characteristics of implementers. Self-efficacy, for example, is a psychological characteristic associated with higher-quality program implementation (Damschroder et al., 2022).

Schools that implement SMH services use screening tools and other available data (e.g., behavioral referrals) to assess and respond to student mental health needs. Schools’ responses to student mental health needs can range from implementing universal prevention programs to interventions targeting small groups or individual students (Christner et al., 2011). Given the range of activities required to effectively implement SMH services (e.g., assessment, prevention programs, targeted interventions), it is possible individuals may vary in their confidence to accomplish these tasks (i.e., self-efficacy) depending on the type of SMH task at hand. Studies on SMH professional development have acknowledged this by investigating targeted professional development interventions that bolster specific types of self-efficacy, such as clinical self-efficacy (Lyons et al., 2023; Taylor et al., 2023).

### Self-Efficacy as a Component of Effective School Mental Health Implementation

Self-efficacy is a concept introduced through Bandura’s *Social Cognitive Theory* (1997) that refers to an individual’s belief in their abilities to accomplish goals or carry out a task (e.g., an intervention). Given the role of self-efficacy in implementation (Damschroder et al., 2022), measuring self-efficacy and its sources may improve implementation of SMH services. Comprehensive SMH services often require collaboration across several school-based professions and involve a variety of roles and responsibilities. As such, self-efficacy in the context of SMH can have multiple meanings depending on the profession and role of the individual. Teachers, for example, play an essential role in providing SMH services and are often involved in implementing universal SMH interventions (Franklin et al., 2012). Further, teachers are often involved in completing assessments to identify students with mental health concerns to SMH professionals (Ekornes, 2015). As teachers are involved in providing SMH services in various ways, they may have differing levels of self-efficacy depending on the task at hand (e.g., recognition and referral). Thus, researchers and school leaders may be able to tailor professional development experiences with psychometrically validated measures of teacher self-efficacy that assess distinct but related aspects of SMH self-efficacy.

### Measurement of School Mental Health Self-Efficacy

There are limited tools available for measuring teacher SMH self-efficacy. As noted by Tschannen-Moran and Hoy (2001), the construct conceptualization of teacher self-efficacy is varied with different theoretical underpinnings. For example, some scales have had a more general, unidimensional conceptualization of self-efficacy (e.g., Teacher Efficacy Scale; Gibson & Dembo, 1984) while others are specific to certain domains of teaching (e.g., Teacher Sense of Efficacy Scale; Tschannen-Moran & Hoy, 2001). As stated by Bandura, teacher self-efficacy is not necessarily uniform across the many roles teachers hold, and specialized scales should be developed that are linked to various knowledge domains (1997).

The SMH-SETS (Brann et al., 2021), the focus of this study, is the only scale available that measures teachers’ beliefs in their capacity to support students’ mental health needs. The scale was initially developed in a sample of 204 preschool through grade 12 general and special education teachers from one rural Midwestern school district. The 15-item measure captures teachers’ attitudes about their ability to recognize, provide academic instruction to, and make referrals for students with mental health needs. The SMH-SETS was originally developed and analyzed through Rasch theory and Rasch analysis. The unidimensionality of items in the scale was determined using multiple methods, including Infit and Outfit Mean Squares (MNSQ) statistics, Principal Component Analysis of Residuals (PCAR), and disattentuated correlation between person measures for each cluster of items (Brann et al., 2021). However, given that the scale was initially deployed in a sample restricted to one school district across grade levels, psychometric investigation in a broader sample of middle schools can provide additional evidence about the construct validity of the scale.

Beyond the initial psychometric study, there is limited published work regarding the psychometric properties of the SMH-SETS. However, there are a few applications of the measure that demonstrate expected relationships with exposure to teacher professional development. In a sample of pre-service teachers, the measure demonstrated sensitivity to training in student mental health, with pre-service teachers receiving student mental health training reporting significantly greater levels of school mental health self-efficacy, notably with a moderate effect size (Brann et al., 2022). In addition, the SMH-SETS has also been used to assess change in teacher self-efficacy in response to teacher professional development programs (Bichoualne et al., 2023; Habgood et al., 2025).

Despite this initial promise, additional evidence is needed regarding the SMH-SETS internal structure and relationships with other variables. A systematic review of SMH measures that suggests the SMH-SETS demonstrates “sufficient” yet “low” overall evidence of structural validity and no evidence of hypothesis tests supporting relationships with other constructs (e.g., convergent validity; Smith et al., 2024) when evaluated using the *Consensus-Based Standards for the Selection of Health Instruments* (Prinsen et al., 2018). Considering past studies that discuss the varied roles of teachers in supporting SMH systems (Ekornes, 2015; Franklin et al., 2012), it may be possible that the SMH-SETS is not a unidimensional construct when applied to other samples. The practices referenced in the SMH-SETS conceptually map onto different SMH competencies, such as recognition of mental health problems, intervention, and cultural competence (Ball et al., 2010). Thus, it is plausible that that SMH-SETS items may be better represented as a multidimensional scale measuring subtypes of self-efficacy for implementing SMH services. Moreover, it will be important to understand how scores from the SMH-SETS relate to other SMH implementation constructs.

### Current Study

The current study seeks to provide additional validity evidence for a scale designed to measure SMH self-efficacy in teachers, the SMH-SETS (Brann et al., 2021), specifically investigating the internal structure of the measure as well as relationships with SMH implementation variables. Self-efficacy is identified in several implementation frameworks as an individual-level characteristic that influences the outcome of implementation. As such, there is a need for psychometrically sound measures that investigate self-efficacy, especially in the context of SMH. For our first research aim, we intend to provide construct validity (American Educational Research Association [AERA] et al., 2014) evidence related to the internal structure of the scale in a sample of middle school teachers. We conducted a series of confirmatory factor analyses to determine whether the SMH-SETS is better captured through a unidimensional structure, as it was originally published, or through a theoretically informed multi-factor structure. Given the myriad SMH demands placed on schoolteachers (Ball et al., 2010), we hypothesized that a multifactor model of SMH-SETS would yield greater fit than a unidimensional model. Second, we explore associations between the SMH-SETS scores and reaction measures of intervention implementation to examine convergent validity of the SMH-SETS (AERA et al., 2014).

## Method

### Procedure

The current study is part of a broader study that centers around testing implementation supports designed to improve SMH services. As part of the larger project, school staff, including teachers, are implementing the *Early Identification System* (EIS; Herman et al., 2023), a universal mental and behavioral health checklist that supports selection and implementation of evidence-based interventions. Implementation of the EIS involves several competencies that are captured through the SMH-SETS (Brann et al., 2021) and are key elements of comprehensive SMH systems (Ball et al., 2010; Zabek et al., 2023), such as identification of mental health concerns and student-family collaboration. This study utilizes data collected from the first two cohorts of schools participating in a multistate group randomized controlled trial. Schools were recruited as part of a larger study, approved by institutional review boards within each participating state, investigating and supporting the implementation of a universal mental and behavioral health checklist, EIS. The current study used pre-intervention data collected across two cohorts enrolled in the larger study. All pre-intervention data was collected in the fall after consenting teachers completed the EIS checklist and before the broader study intervention began.

### Participants

This study utilizes pre-intervention data from two consecutive years of implementation, 2023–2024 and 2024–2025. The data reflects two cohorts of participants from a total of 32 schools and 29 districts in predominantly small city and rural locales(NCES, n.d.2025) across three southern and midwestern states that completed baseline measures prior to receiving the intervention. Participants (*N* = 411) include general education teachers (*n* = 350), special education teachers (*n* = 50), and other teachers, such as elective teachers (*n* = 11). All teachers taught within the middle school grades (i.e., grades 6–8). Additional participant demographics are presented in Table 1, and school-level characteristics are reported in the Appendix.

### Measures

#### School Mental Health Self-Efficacy

Participant self-efficacy in supporting student mental health was measured through the SMH-SETS (Brann et al., 2021). An example from the 15-item scale includes “I feel confident in my ability to discuss student mental health concerns with parents/guardians.” Participants responded on a 6-point Likert scale (1 = *Strongly disagree* to 6 = *Strongly agree*). Factor scores were extracted from a confirmatory factor analytic model where higher scores indicated greater perceived self-efficacy. Table 2 contains item-level descriptions, means, and standard deviations for all 15 items. Higher item-level means may be interpreted as the respondent feeling more efficacious in an aspect of SMH (e.g., recognizing a student with an externalizing concern).

#### Intervention Reaction Measures

Participant’s reaction to the larger study’s universal mental and behavioral health screening system, EIS, was measured through three, four-item implementation outcome measures established by Weiner et al. (2017), the *Acceptability of Intervention Measure* (AIM), *Intervention Appropriateness Measure* (IAM), and *Feasibility of Intervention Measure* (FIM). Examples from each measure include “The EIS meets my approval,” “The EIS seems fitting,” and “The EIS seems implementable,” respectively. Participants responded on a 5-point Likert scale (1 = *Completely disagree* to 5 = *Completely agree*) for each measure. A score for the three measures was obtained by taking the average of each measure. Cronbach’s alpha indicated excellent internal consistency across the AIM (α = .90), IAM (α = .96), and FIM (α = .93) measures.

### Data Analysis

We utilized confirmatory factor analysis (CFA) to provide evidence of internal structure. CFA model fit was assessed through the Comparative Fit Index (CFI), Tucker-Lewis Index (TLI), Root Mean Square Error of Approximation (RMSEA), and Standardized Root Mean Squared Residual (SRMR). Values of CFI ≥ 0.95, TLI ≥ 0.95, RMSEA ≤ 0.06, and SRMR ≤ 0.08 indicated good fit between the hypothesized model and observed data (Hu & Bentler, 1999). Weighted Least Square Mean and Variance adjusted (WLSMV) estimators were used due to the non-normal distribution of responses and ordinal nature of the SMH-SETS (Li, 2016). Based on guidance from DiStefano et al. (2021), we reviewed the distribution of item responses and collapsed across response categories to ensure that each response level meets a minimum of 40 responses, or roughly 10% of the sample.

We initially fit a full model with all 15 items loading on one latent factor, SMH self-efficacy, in accordance with the unidimensional structure proposed by the SMH-SETS authors (Brann et al., 2021). We compared this one factor model to a two factor and five factor model that were based on theoretically informed subdimensions. The two-factor model divided self-efficacy items into academic (seven items) and behavior-focused subdomains (eight items). The five-factor model further differentiated domains of SMH self-efficacy into recognition of difficulties among students (Recognition), academic instruction (Academic), considering needs of diverse students (Diversity), responding when a student is demonstrating acute behavioral health difficulties (Crisis), and providing general and universal SMH supports (e.g., referral, coordination with caregivers, universal mental health classroom-based supports; Support). Our partitioning of the SMH-SETS into two- and five-factor structures was based on findings from systematic reviews illustrating the domains involved in SMH systems (Ball et al., 2010; Zabek et al., 2023) as well as evidence suggesting teachers’ differentiated roles in SMH, such as classroom intervention and referral (Ekornes, 2015; Franklin et al., 2012).

Next, to assess validity evidence based on relationships with other constructs, we fit ordinary least squares (OLS) fixed-effects regression models to explore the association between SMH-SETS extracted factor scores and AIM, IAM, and FIM scores. Each factor from the best fitting model was entered into the regression model as a covariate, and a model was computed for each of the three intervention reaction scales. School-level variance was controlled for by including school as a covariate.

All analyses were conducted using R Statistical Software (v4.4.2; R Core Team, 2024). CFAs were conducted using the package *lavaan* (v0.6.19; Rosseel, 2012). OLS regressions were conducted using base R.

## Results

**Fig. 1:**
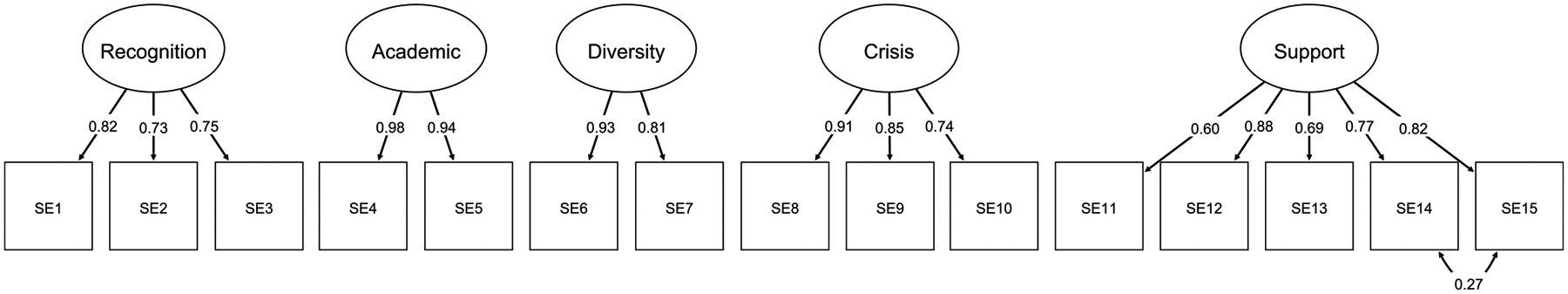
Modified five-factor CFA model. All factor loadings are standardized and significant at *p* ≤ .001. Additionally, all factors were allowed to correlate. See Table 4 for correlations between factors. See Table 2 for item-level descriptions

We first fit a one-factor CFA model that assumed all 15 scale items loaded onto a single “SMH self-efficacy” factor. The one-factor model demonstrated poor fit, χ^2^ (90) = 1030.98, *p* < .001. The CFI (.89), TLI (.87), RMSEA (.16), and SRMR (.13) failed to meet their respective cutoffs, indicating poor model-data fit. Next, both two-factor and five-factor models were evaluated. Table 3 contains fit information for all models. The two-factor solution demonstrated improvement compared to the one-factor model as measured by the scaled chi-square difference test, Δχ^2^ (1) = 113.94, *p* < .001; however, the CFI (.94), TLI (.93), RMSEA (.12), and SRMR (.09) failed to meet their respective cutoffs. The five-factor solution demonstrated considerable improvement over the two-factor model, Δχ^2^ (9) = 201.53, *p* < .001. While the CFI (.98), TLI (.97), and SRMR (.05) met their respective cutoffs, the RMSEA (.08) was above the .06 cutoff. Based on modification indices for the five-factor model, a correlated error term between items 14 and 15 was added to improve model fit. This adjustment was theoretically justified as these items may share measurement error due to similar wording and content overlap. After incorporating this modification, the modified five-factor model demonstrated improved fit compared to the unmodified five-factor model, Δχ^2^ (1) = 51.78, *p* < .001. The CFI (.99), TLI (.99), RMSEA (.05), and SRMR (.04) all met their respective cutoffs, indicating the modified five-factor model as the best fitting model. The modified five-factor model with standardized factor loadings is shown in Fig. 1. Table 4 contains a scaled covariance matrix of the factors, interpretable as the correlation, and indicates a positive relationship between all factors. Latent factor scores for the final model were extracted to explore our secondary research aim.

OLS regressions explored the relationship of the five SMH-SETS factor scores with three EIS reaction measures: AIM, IAM, and FIM. Regression results are displayed in Table 5. The model predicting EIS acceptability, AIM (*M* = 3.57, *SD* = 0.64), was statistically significant, *F* (36, 374) = 2.71, *p* < .001, with an adjusted *R*^2^ of .13. Of the five factors, Crisis (β = −.32, *p* = .02) and Support (β = .46, *p* = .01) were significant predictors of AIM. The model predicting EIS appropriateness, IAM (*M* = 3.70, *SD* = 0.67), was statistically significant, *F* (36, 374) = 1.75, *p* = .01, with an adjusted *R*^2^ of .06. Of the five factors, Support (β = .39, *p* = .02) was a significant predictor of IAM. The model predicting EIS feasibility, FIM (*M* = 3.67, *SD* = 0.66), was statistically significant, *F* (36, 374) = 2.09, *p* < .001, with an adjusted *R*^2^ of .09. Of the five factors, Support (β = .42, *p* = .01) was a significant predictor of FIM.

## Discussion

The current study investigated the validity of the SMH-SETS (Brann et al., 2021) in a sample of middle school teachers by testing alternative factor structures and relationships among subscales and with other variables (i.e., teachers’ reactions to the implementation of the EIS). On average, at the item-level, respondents tended to “somewhat agree” or “agree” to being efficacious in various aspects of SMH. This is roughly consistent with prior studies utilizing the SMH-SETS (e.g., Brann et al., 2022; Habgood et al., 2025). Overall, our findings indicated that a unidimensional model of SMH self-efficacy did not fit the data used in this study. Rather, a five-factor model provided the best fit for our data. We also tested associations between the five-factor SMH-SETS scale and teachers’ reactions to the implementation of the EIS, a universal mental and behavioral health checklist. A differential pattern of relationships suggests that assessment of SMH self-efficacy using a multifactor measure of self-efficacy may permit researchers and school leaders to tailor professional development efforts to teachers’ training needs. In addition, the positive correlations between the SMH-SETS scale and teachers’ reactions to the EIS offers additional insight into how subdomains of self-efficacy relate to implementation of a mental and behavioral health checklist.

Our initial analysis tested the unidimensional structure of the SMH-SETS. Owing to poor fit, we then tested theoretically driven, multi-factor structures of the measure with the hypothesis that they would yield greater fit than a one-factor solution. A two-factor model with Academic and Mental Health factors, while conceptually appealing and an improvement from the one-factor model, did not meet conventional fit criteria. A five-factor model demonstrated considerable improvement compared to the two-factor model while meeting established thresholds on most fit criteria, and a minor modification to the five-factor model demonstrated the best fit. The factors in the best fitting model were labeled Recognition, Academic, Diversity, Crisis, and Support, and are discussed next.

The subconstructs within the five-factor model were developed from an understanding that the items within the SMH-SETS conceptually aligned with different skills and competencies required for implementing evidence-based SMH services (Ball et al., 2010). First (1), items in the Recognition factor involved identifying students who have a mental health concern. Second (2), items in the Crisis factor involve responding to students who have an acute mental health need. Both Recognition and Crisis factors are a core element of screening, intervention, and monitoring procedures in SMH (Ball et al., 2010). Third (3), the Academic factor involved rendering academic services to students with mental health concerns and can be likened to competencies relating to adapting curricula and instruction to students’ needs. The Recognition, Crisis, and Academic factors overlap specifically with “Provision of academic, social-emotional, and behavioral learning supports” identified by Ball et al. (2010). Fourth (4), the Diversity factor overlapped with “Cultural competence” and involves providing culturally appropriate academic or behavioral services to students from diverse backgrounds (Ball et al., 2010). Fifth (5), the Support factor spans multiple competencies, such as interprofessional collaboration, engagement with multiple systems (e.g., families), and classroom-based mental health promotion (Ball et al., 2010). As expected, these subscales were all positively correlated but the pattern of relationships suggested that the scales were differentiating, with all but one correlation below .8.

Next, we examined how the five factors of the SMH-SETS were associated with teacher ratings of acceptability, appropriateness, and feasibility of using the EIS. Importantly, the five factor scores had a different pattern of relationships with EIS acceptability, appropriateness, and feasibility, providing additional evidence that the subconstructs are differentiated and important to assess individually. Consistent with our hypotheses, we found that the Support factor was significantly associated with teacher ratings of EIS acceptability (β = .46), appropriateness of the intervention (β = .39), and feasibility of the EIS (β = .42). The Support factor references a greater set of skills, such as referrals, family collaboration, and classroom-based mental health promotion. Many of the skills identified within the Support factor are leveraged in Tier 1 and Tier 2 interventions (Stoiber & Gettinger, 2016), which is the domain EIS targets the most. Contrary to our hypotheses, we found that the Crisis factor was significantly and negatively associated with teacher ratings of EIS acceptability (β = −.32). No other correlation was found to be significant; however, small and negative effect sizes were also observed for the EIS appropriateness and feasibility measures. The negative association between the Crisis factor and EIS acceptability may suggest that teachers with greater self-efficacy in navigating students’ acute mental health needs found the intervention to be less acceptable. It is possible that factors such as school resources or access to other SMH personnel may moderate this relationship (Palmer et al., 2025).

### Limitations and Future Directions

Although findings add to the literature on the measurement of middle school teachers’ SMH self-efficacy, it is not without limitations. Our study consisted of middle school teachers from predominantly rural southern and midwestern states in the United States. Factor analysis findings support a five-factor solution to our data; however, we wish to emphasize that the scale may function differently in different samples. For example, cultural and contextual factors may influence individuals’ perceptions of self-efficacy (Scholz et al., 2002) as well as produce variation in opportunities for pursuing mastery and vicarious experiences (Bandura, 1997). As our sample consisted solely of middle school teachers, results may not generalize to elementary or high school teachers, who interface with students of different development stages and may interpret students’ mental and behavioral health differently (Green et al., 2022). Investigating and comparing the use of SMH-SETS with teachers from various grade levels may lead to implications for grade-specific measurement. Future research should compare the internal structure of the measure across grades and consider differential item functioning. Additionally, we administered this scale with instructions and items as originally developed and could not evaluate teacher response processes in the current study. Additional validity evidence is needed regarding teacher response processes when completing the SMH-SETS to better understand how teachers are interpreting and responding to specific items (e.g., differentiating “internalizing” vs. “externalizing” problems).

Furthermore, we depended on cross-sectional self-report data to explore our secondary research question, limiting our ability to make causal influences about the relationship between facets of self-efficacy and teachers’ reactions to EIS implementation. Future studies may wish to involve a multi-method approach, such as observational or performance-based assessments, to achieve a more triangulated understanding of teachers’ SMH self-efficacy. Researchers may also wish to perform additional convergent or predictive validity checks, such as evaluating how self-efficacy measured through the SMH-SETS compares to or discriminates from other broader measures of teacher self-efficacy (e.g., Tschannen-Moran & Hoy, 2001). Additionally, while the identified five-factor structure of the SMH-SETS aligns with professional SMH competencies identified by Ball et al. (2010), there are several conceptually relevant competencies that are not reflected in the scale (e.g., understanding of policies and laws, interprofessional collaboration). This may be a potential opportunity for the development of a SMH self-efficacy scale that captures more facets of SMH service delivery. Last, our finding that the Crisis factor of the five-factor SMH-SETS model was significantly and negatively associated with teacher ratings of EIS acceptability warrants additional investigation. This presents an opportunity for future research to investigate how variables not investigated within the current study, such as teachers’ years in the profession, access to school resources, and other roles held, impact associations between SMH self-efficacy and the perceived acceptability of universal mental health screening (e.g., EIS).

### Implications for School Mental Health

The modified five-factor structure of the SMH-SETS offers a more detailed understanding of the components of teachers’ SMH self-efficacy. First, it may help both researchers and practitioners to conceptualize SMH self-efficacy as a multi-factor construct. Following this conceptualization, it is possible for researchers to measure certain facets of SMH self-efficacy, such as self-efficacy in recognizing student mental health concerns. It can also assist intervention and professional development efforts by providing targets for developing teachers’ self-efficacy in a particular area. Second, the consistent predictive value of the Support factor across intervention reaction outcomes suggests that efforts to enhance school-family collaboration and classroom-based promotion may be of particular interest when implementing SMH screening initiatives. Overall, the findings of the current study highlight the value of using a multidimensional approach to assessing teachers’ self-efficacy as a mechanism for strengthening SMH systems is represented as the average percentage of students across schools

## Figures and Tables

**Table 1:** Sample demographics

	*n* (%)
*Gender*	
Female	308 (74.94)
Male	91 (22.14)
Other	12 (2.92)
*Race*	
Black/African American	14 (3.41)
White/European American	387 (94.16)
Other	10 (2.43)
*Level of Education*	
Bachelor’s degree	192 (46.72)
Master’s degree	191 (46.47)
Specialist degree	14 (3.41)
Other	14 (3.41)
*School Role*	
General education teacher	350 (85.16)
Special education teacher	50 (12.17)
Other teacher	11 (2.68)
*Years of Experience in Teaching Role*	
< 1 year	25 (6.08)
1–2 years	38 (9.25)
3–5 years	84 (20.44)
6–10 years	83 (20.19)
11–20 years	109 (26.52)
> 20 years	72 (17.52)
Total	411

*Note*. Demographic categories with small cell sizes (< 10) were collapsed into an “Other” category to protect participant confidentiality and ensure anonymity

**Table 2:** Item-level descriptions, means, and standard deviations for the SMH-SETS

I feel confident in my ability to …	Mean	SD	Factor
1. recognize when there is a student with an internalizing concern	4.54	0.78	Recognition
2. recognize when there is a student with an externalizing concern	5.00	0.71	Recognition
3. recognize when there is a student displaying indicators of exposure to trauma	4.57	0.82	Recognition
4. provide academic instruction to students with an internalizing concern	4.55	0.74	Academic
5. provide academic instruction to students with an externalizing concern	4.59	0.80	Academic
6. provide academic instruction to students with diverse backgrounds who have mental health concerns	4.74	0.81	Diversity
7. consider cultural needs in promoting students’ mental health	4.79	0.78	Diversity
8. respond when a student is in crisis	4.87	0.89	Crisis
9. respond when a student is displaying aggressive behavior	4.73	0.94	Crisis
10. respond to a student who is expressing suicidal thoughts	4.64	1.02	Crisis
11. refer a student to the appropriate school-based mental health providers	5.18	0.78	Support
12. offer assistance in the classroom when a student is struggling with a mental health concern	4.76	0.89	Support
13. discuss student mental health concerns with parents/guardians	4.08	1.18	Support
14. promote the social skills of students in my classroom	5.02	0.76	Support
15. promote the emotional skills of students in my classroom	4.91	0.80	Support

*Note*. Participants responded on a 6-point Likert scale (1 = *Strongly disagree* to 6 = *Strongly agree*). The means and standard deviations reported above were computed prior to collapsing response categories to ensure at least 10% of responses in each category. Each item’s corresponding factor according to the final five-factor model is also reported

**Table 3:** Fit information for CFA models

Model	χ^2^	*df*	CFI	TLI	RMSEA (90% CI)	SRMR
Unidimensional	1030.98	90	.89	.87	.16 (.15—.17)	.13
Two-factor	616.33	89	.94	.93	.12 (.11—.13)	.09
Five-factor	283.43	80	.98	.97	.08 (.07—.09)	.05
Modified five-factor	159.90	79	.99	.99	.05 (.04—.06)	.04

*Note*. Scaled versions of the χ^2^, Comparative Fit Index (CFI), Tucker-Lewis Index (TLI), Root Mean Square Error of Approximation (RMSEA), and Standardized Root Mean Squared Residual (SRMR) are reported. All χ^2^ model tests were statistically significant at *p* ≤ .001

**Table 4:** Scaled covariance matrix of the modified five-factor CFA model

Factor	1	2	3	4	5
1. Recognition	–				
2. Academic	.71	–			
3. Diversity	.71	.68	–		
4. Crisis	.71	.52	.56	–	
5. Support	.76	.53	.70	.85	–

*Note*. Factors were scaled to have a variance of one, and their covariances may be interpreted as the correlation

**Table 5:** OLS Regressions on EIS reaction measures

Predictor	AIM		IAM		FIM	
	β	SE	β	SE	β	SE
Recognition	.09	0.11	.03	0.12	.05	.11
Academic	.15	0.10	.15	0.11	.03	.11
Diversity	−.06	0.08	−.09	0.09	.00	.09
Crisis	−.32*	0.10	−.24	0.10	−.23	.10
Support	.46**	0.12	.39*	0.13	.42*	.12

Note

** *p* ≤ .01;

* *p* ≤ .05.

Standardized beta coefficients are reported. School-level clustering was accounted for by including school as a covariate; however, due to the number of schools, their coefficients are not listed. Early Identification System (EIS) reaction measures include the Acceptability of Intervention Measure (AIM), Intervention Appropriateness Measure (IAM), and Feasibility of Intervention Measure (FIM)

## Appendix

**Table:** School-Level Characteristics.

School Characteristic	M	SD	*n*
Total Enrollment	469.75	262.02	24
*Student Racial/Ethnic Makeup (%)*			
American Indian	0.41	0.48	12
Asian	1.06	0.76	20
Black	14.75	11.71	21
Hawaiian or Pacific Islander	0.15	0.21	10
Hispanic	7.60	7.41	22
White	73.63	18.28	24
Two or More Races	6.02	3.45	21
Free or Reduced-Price Lunch (%)	54.53	22.29	18
Student-Teacher Ratio	13.73	2.04	24

*Note*. Demographic and school-level data is taken from the NCES. The study includes 32 schools; however, data is not available for all schools. Means (M), standard deviations (SD), and the total valid cases (*n*) are reported. Student racial/ethnic makeup and free or reduced-price lunch status
